# 2-(4-Bromo­phen­yl)-5-fluoro-3-phenyl­sulfinyl-1-benzofuran

**DOI:** 10.1107/S1600536810029958

**Published:** 2010-07-31

**Authors:** Hong Dae Choi, Pil Ja Seo, Byeng Wha Son, Uk Lee

**Affiliations:** a Department of Chemistry, Dongeui University, San 24 Kaya-dong Busanjin-gu, Busan 614-714, Republic of Korea; b Department of Chemistry, Pukyong National University, 599-1 Daeyeon 3-dong, Nam-gu, Busan 608-737, Republic of Korea

## Abstract

In the title compound, C_20_H_12_BrFO_2_S, the O atom and the phenyl group of the phenyl­sulfinyl substituent lie on opposite sides of the plane through the benzofuran fragment; the phenyl ring is nearly perpendicular to this plane [dihedral angle = 86.98 (6)°]. The 4-bromo­phenyl ring is rotated slightly out of the benzofuran plane, making a dihedral angle of 1.56 (8)°. The crystal structure features aromatic π–π inter­actions between the furan and phenyl rings of neighbouring mol­ecules [centroid–centroid distance = 3.506 (3) Å], and an inter­molecular C—H⋯π inter­action. The crystal structure also exhibits a short inter­molecular S⋯S contact [3.2635 (8) Å].

## Related literature

For the pharmacological activity of benzofuran compounds, see: Aslam *et al.* (2006 ▶); Galal *et al.* (2009 ▶); Khan *et al.* (2005 ▶). For natural products with benzofuran rings, see: Akgul & Anil (2003 ▶); Soekamto *et al.* (2003 ▶). For the structures of related 5-halo-2-phenyl-3-phenyl­sulfinyl-1-benzofuran derivatives, see: Choi *et al.* (2009**a* ▶,*b* ▶,c* ▶). For short S⋯S inter­actions, see: Munshi & Guru Row (2004 ▶).
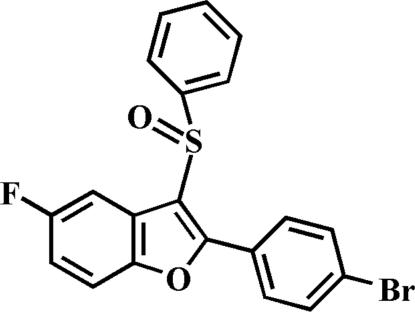


## Experimental

### 

#### Crystal data

C_20_H_12_BrFO_2_S
*M* *_r_* = 415.27
Triclinic, 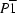

*a* = 8.1361 (4) Å
*b* = 9.8237 (5) Å
*c* = 11.4093 (5) Å
α = 82.866 (3)°
β = 77.123 (3)°
γ = 69.155 (2)°
*V* = 829.78 (7) Å^3^
*Z* = 2
Mo *K*α radiation
μ = 2.62 mm^−1^
*T* = 173 K
0.29 × 0.26 × 0.21 mm

#### Data collection

Bruker SMART APEXII CCD diffractometer
Absorption correction: multi-scan (*SADABS*; Bruker, 2009 ▶) *T*_min_ = 0.664, *T*_max_ = 0.746
14582 measured reflections
3833 independent reflections
3452 reflections with *I* > 2σ(*I*)
*R*_int_ = 0.033

#### Refinement

*R*[*F* ^2^ > 2σ(*F* ^2^)] = 0.030
*wR*(*F* ^2^) = 0.104
*S* = 0.87
3833 reflections
226 parameters
H-atom parameters constrained
Δρ_max_ = 0.37 e Å^−3^
Δρ_min_ = −0.63 e Å^−3^

### 

Data collection: *APEX2* (Bruker, 2009 ▶); cell refinement: *SAINT* (Bruker, 2009 ▶); data reduction: *SAINT*; program(s) used to solve structure: *SHELXS97* (Sheldrick, 2008 ▶); program(s) used to refine structure: *SHELXL97* (Sheldrick, 2008 ▶); molecular graphics: *ORTEP-3* (Farrugia, 1997 ▶) and *DIAMOND* (Brandenburg, 1998 ▶); software used to prepare material for publication: *SHELXL97*.

## Supplementary Material

Crystal structure: contains datablocks global, I. DOI: 10.1107/S1600536810029958/pk2256sup1.cif

Structure factors: contains datablocks I. DOI: 10.1107/S1600536810029958/pk2256Isup2.hkl

Additional supplementary materials:  crystallographic information; 3D view; checkCIF report

## Figures and Tables

**Table 1:** Hydrogen-bond geometry (Å, °) *Cg*3 is the centroid of the C15–C20 phenyl ring.

*D*—H⋯*A*	*D*—H	H⋯*A*	*D*⋯*A*	*D*—H⋯*A*
C5—H5⋯*Cg*3^i^	0.93	2.85	3.644 (3)	145

Symmetry code: (i) 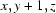.

**Fig. 1.**
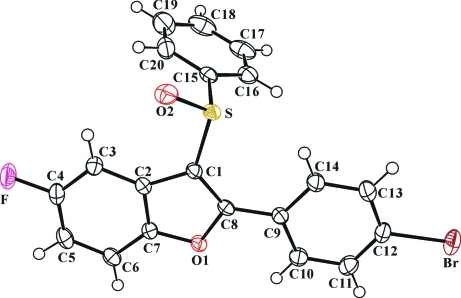
The molecular structure of the title compound with the atom numbering scheme. Displacement ellipsoids are drawn at the 50% probability level. H atoms are presented as a small spheres of arbitrary radius.

**Fig. 2.**
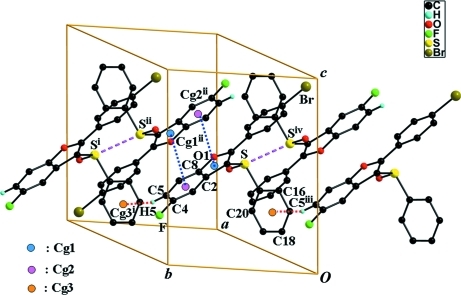
π–π, C—H···π and S···S interactions (dotted lines) in the crystal structure of the title compound. Cg denotes the ring centroids. [Symmetry codes: (i) x, y + 1, z; (ii) - x + 1, - y + 1, - z + 1; (iii) x, y - 1, z; (iv) - x + 1, - y, - z + 1.]

## supplementary crystallographic information

### Comment

Many compounds containing a benzofuran moiety have attracted considerable interest in view of their pharmacological properties such as antifungal, antitumor and antiviral, antimicrobial activities (Aslam *et al.*, 2006, Galal *et al.*, 2009, Khan *et al.*, 2005). These compounds occur widely in nature (Akgul & Anil, 2003; Soekamto *et al.*, 2003). As a part of our continuing studies of the substituent effect on the solid state structures of 5-halo-2-phenyl-3-phenylsulfinyl-1-benzofuran analogues (Choi *et al.*, 2009**a*,*b*,c*), we report the crystal structure of the title compound (Fig. 1).

The benzofuran unit is essentially planar, with a mean deviation of 0.063 (1) Å from the least-squares plane defined by the nine constituent atoms. The dihedral angle formed by the benzofuran plane and the phenyl ring is 86.98 (6)°, and the 4-bromophenyl ring with 1.56 (8)° lies toward the benzofuran plane. The crystal packing (Fig. 2) is stabilized by aromatic π–π interactions between the furan and benzene rings of the adjacent molecules, with a Cg1···Cg2^ii^ distance of 3.506 (3) Å (Cg1 and Cg2 are the centroids of the C1/C2/C7/O1/C8 furan ring and the C2–C7 benzene ring, respectively). The molecular packing (Fig. 2) is further stabilized by an intermolecular C—H···π interaction between the benzene H atom and the phenyl ring of a neighbouring molecule, with a C5—H5···Cg3^i^ (Table 1; Cg3 is the centroid of the C15–C20 phenyl ring). The crystal structure also exhibits a short intermolecular S···S contact (Munshi & Guru Row, 2004), with a S···S^iv^ distance of 3.2635 (8) Å

### Experimental

77% 3-chloroperoxybenzoic acid (247 mg, 1.1 mmol) was added in small portions to a stirred solution of 2-(4-bromophenyl)-5-fluoro-3-phenylsulfanyl-1-benzofuran (439 mg, 0.8 mmol) in dichloromethane (40 mL) at 273 K. After being stirred at room temperature for 6h, the mixture was washed with saturated sodium bicarbonate solution and the organic layer was separated, dried over magnesium sulfate, filtered and concentrated at reduced pressure. The residue was purified by column chromatography (hexane-ethyl acetate, 4:1 v/v) to afford the title compound as a colorless solid [yield 68%, m.p. 465–466 K; *R*_f_ = 0.75 (hexane–ethyl acetate, 4:1 v/v)]. Single crystals suitable for X-ray diffraction were prepared by slow evaporation of a solution of the title compound in benzene at room temperature.

### Refinement

All H atoms were positioned geometrically and refined using a riding model, with C—H = 0.93 Å and *U*_iso_(H) = 1.2*U*_eq_(C).

### Crystal data

**Table:**

C_20_H_12_BrFO_2_S	*Z* = 2
*M**_r_* = 415.27	*F*(000) = 416
Triclinic, *P*1	*D*_x_ = 1.662 Mg m^−^^3^
Hall symbol: -P 1	Mo *K*α radiation, λ = 0.71073 Å
*a* = 8.1361 (4) Å	Cell parameters from 8238 reflections
*b* = 9.8237 (5) Å	θ = 2.7–27.5°
*c* = 11.4093 (5) Å	µ = 2.62 mm^−^^1^
α = 82.866 (3)°	*T* = 173 K
β = 77.123 (3)°	Block, colourless
γ = 69.155 (2)°	0.29 × 0.26 × 0.21 mm
*V* = 829.78 (7) Å^3^	

### Data collection

**Table:**

Bruker SMART APEXII CCD diffractometer	3833 independent reflections
Radiation source: rotating anode	3452 reflections with *I* > 2σ(*I*)
graphite multilayer	*R*_int_ = 0.033
Detector resolution: 10.0 pixels mm^-1^	θ_max_ = 27.6°, θ_min_ = 1.8°
φ and ω scans	*h* = −10→10
Absorption correction: multi-scan (*SADABS*; Bruker, 2009)	*k* = −12→12
*T*_min_ = 0.664, *T*_max_ = 0.746	*l* = −14→14
14582 measured reflections	

### Refinement

**Table:**

Refinement on *F*^2^	Primary atom site location: structure-invariant direct methods
Least-squares matrix: full	Secondary atom site location: difference Fourier map
*R*[*F*^2^ > 2σ(*F*^2^)] = 0.030	Hydrogen site location: difference Fourier map
*wR*(*F*^2^) = 0.104	H-atom parameters constrained
*S* = 0.87	*w* = 1/[σ^2^(*F*_o_^2^) + (0.1*P*)^2^] where *P* = (*F*_o_^2^ + 2*F*_c_^2^)/3
3833 reflections	(Δ/σ)_max_ = 0.001
226 parameters	Δρ_max_ = 0.37 e Å^−^^3^
0 restraints	Δρ_min_ = −0.63 e Å^−^^3^

### Special details

**Table:**

Geometry. All esds (except the esd in the dihedral angle between two l.s. planes) are estimated using the full covariance matrix. The cell esds are taken into account individually in the estimation of esds in distances, angles and torsion angles; correlations between esds in cell parameters are only used when they are defined by crystal symmetry. An approximate (isotropic) treatment of cell esds is used for estimating esds involving l.s. planes.
Refinement. Refinement of F^2^ against ALL reflections. The weighted R-factor wR and goodness of fit S are based on F^2^, conventional R-factors R are based on F, with F set to zero for negative F^2^. The threshold expression of F^2^ > 2sigma(F^2^) is used only for calculating R-factors(gt) etc. and is not relevant to the choice of reflections for refinement. R-factors based on F^2^ are statistically about twice as large as those based on F, and R- factors based on ALL data will be even larger.

### Fractional atomic coordinates and isotropic or equivalent isotropic displacement parameters (Å^2^)

**Table:**

	*x*	*y*	*z*	*U*_iso_*/*U*_eq_	
Br	−0.21990 (3)	0.22528 (2)	0.998902 (16)	0.03781 (11)	
S	0.47916 (5)	0.16534 (4)	0.44461 (4)	0.02033 (12)	
F	0.64982 (17)	0.61956 (15)	0.12311 (11)	0.0381 (3)	
O1	0.17961 (16)	0.57103 (13)	0.53100 (11)	0.0210 (3)	
O2	0.66591 (17)	0.14226 (15)	0.37795 (13)	0.0302 (3)	
C1	0.3705 (2)	0.35537 (18)	0.46114 (14)	0.0193 (3)	
C2	0.4126 (2)	0.46848 (19)	0.37985 (15)	0.0196 (3)	
C3	0.5388 (2)	0.4728 (2)	0.27522 (16)	0.0238 (4)	
H3	0.6234	0.3885	0.2413	0.029*	
C4	0.5298 (2)	0.6096 (2)	0.22567 (16)	0.0264 (4)	
C5	0.4074 (3)	0.7387 (2)	0.27342 (17)	0.0280 (4)	
H5	0.4087	0.8278	0.2353	0.034*	
C6	0.2835 (2)	0.7350 (2)	0.37772 (16)	0.0245 (4)	
H6	0.2004	0.8197	0.4121	0.029*	
C7	0.2906 (2)	0.59795 (19)	0.42783 (15)	0.0201 (3)	
C8	0.2314 (2)	0.42188 (17)	0.55135 (15)	0.0191 (3)	
C9	0.1288 (2)	0.37299 (19)	0.65891 (15)	0.0203 (3)	
C10	−0.0123 (3)	0.4752 (2)	0.73078 (17)	0.0273 (4)	
H10	−0.0387	0.5740	0.7092	0.033*	
C11	−0.1131 (3)	0.4322 (2)	0.83305 (18)	0.0307 (4)	
H11	−0.2057	0.5011	0.8804	0.037*	
C12	−0.0740 (2)	0.2854 (2)	0.86369 (16)	0.0256 (4)	
C13	0.0653 (3)	0.1814 (2)	0.79636 (17)	0.0293 (4)	
H13	0.0911	0.0830	0.8194	0.035*	
C14	0.1662 (3)	0.2245 (2)	0.69446 (17)	0.0275 (4)	
H14	0.2600	0.1546	0.6488	0.033*	
C15	0.3563 (2)	0.14738 (19)	0.33775 (17)	0.0217 (3)	
C16	0.1896 (2)	0.1320 (2)	0.37783 (19)	0.0289 (4)	
H16	0.1418	0.1280	0.4597	0.035*	
C17	0.0958 (3)	0.1228 (2)	0.2932 (2)	0.0396 (5)	
H17	−0.0171	0.1142	0.3183	0.047*	
C18	0.1684 (3)	0.1263 (2)	0.1723 (2)	0.0432 (6)	
H18	0.1041	0.1204	0.1164	0.052*	
C19	0.3370 (4)	0.1387 (3)	0.1332 (2)	0.0461 (6)	
H19	0.3856	0.1406	0.0514	0.055*	
C20	0.4327 (3)	0.1481 (2)	0.21686 (18)	0.0347 (5)	
H20	0.5465	0.1549	0.1918	0.042*	

### Atomic displacement parameters (Å^2^)

**Table:**

	*U*^11^	*U*^22^	*U*^33^	*U*^12^	*U*^13^	*U*^23^
Br	0.03745 (16)	0.05065 (18)	0.02458 (14)	−0.02139 (12)	0.00266 (9)	0.00468 (10)
S	0.0185 (2)	0.0151 (2)	0.0247 (2)	−0.00334 (16)	−0.00247 (16)	−0.00170 (16)
F	0.0399 (7)	0.0428 (7)	0.0292 (6)	−0.0205 (6)	0.0043 (5)	0.0072 (5)
O1	0.0219 (6)	0.0140 (6)	0.0237 (6)	−0.0041 (5)	−0.0017 (5)	0.0004 (4)
O2	0.0171 (6)	0.0280 (7)	0.0422 (8)	−0.0040 (5)	−0.0009 (5)	−0.0096 (6)
C1	0.0199 (8)	0.0158 (8)	0.0213 (8)	−0.0059 (6)	−0.0031 (6)	0.0000 (6)
C2	0.0205 (8)	0.0188 (8)	0.0211 (8)	−0.0085 (7)	−0.0054 (6)	0.0007 (6)
C3	0.0226 (8)	0.0260 (9)	0.0225 (8)	−0.0097 (7)	−0.0014 (7)	−0.0012 (7)
C4	0.0268 (9)	0.0325 (10)	0.0226 (8)	−0.0159 (8)	−0.0036 (7)	0.0044 (7)
C5	0.0328 (10)	0.0262 (10)	0.0292 (9)	−0.0154 (8)	−0.0113 (8)	0.0095 (7)
C6	0.0265 (9)	0.0177 (8)	0.0290 (9)	−0.0069 (7)	−0.0079 (7)	0.0025 (7)
C7	0.0202 (8)	0.0198 (8)	0.0205 (8)	−0.0074 (7)	−0.0045 (6)	0.0008 (6)
C8	0.0201 (8)	0.0133 (8)	0.0235 (8)	−0.0047 (6)	−0.0058 (6)	0.0002 (6)
C9	0.0198 (8)	0.0192 (8)	0.0221 (8)	−0.0072 (7)	−0.0037 (6)	−0.0007 (6)
C10	0.0278 (9)	0.0198 (9)	0.0285 (9)	−0.0040 (7)	−0.0009 (7)	−0.0012 (7)
C11	0.0274 (9)	0.0302 (10)	0.0283 (9)	−0.0060 (8)	0.0027 (7)	−0.0054 (8)
C12	0.0261 (8)	0.0323 (10)	0.0191 (8)	−0.0131 (8)	−0.0015 (7)	0.0015 (7)
C13	0.0345 (10)	0.0234 (9)	0.0283 (9)	−0.0114 (8)	−0.0029 (8)	0.0040 (7)
C14	0.0279 (9)	0.0197 (9)	0.0291 (9)	−0.0061 (7)	0.0024 (7)	−0.0010 (7)
C15	0.0215 (8)	0.0150 (8)	0.0283 (9)	−0.0054 (7)	−0.0053 (7)	−0.0012 (6)
C16	0.0215 (9)	0.0227 (9)	0.0403 (11)	−0.0065 (7)	−0.0011 (8)	−0.0057 (8)
C17	0.0263 (9)	0.0240 (10)	0.0699 (15)	−0.0047 (8)	−0.0149 (10)	−0.0091 (10)
C18	0.0558 (14)	0.0281 (11)	0.0552 (14)	−0.0124 (10)	−0.0337 (12)	−0.0003 (10)
C19	0.0761 (17)	0.0456 (14)	0.0295 (11)	−0.0333 (13)	−0.0178 (11)	0.0040 (10)
C20	0.0426 (12)	0.0387 (12)	0.0288 (10)	−0.0246 (10)	−0.0018 (9)	−0.0005 (8)

### Geometric parameters (Å, °)

**Table:**

Br—C12	1.8961 (18)	C9—C14	1.406 (2)
S—S^i^	3.2635 (8)	C10—C11	1.382 (3)
S—O2	1.4903 (13)	C10—H10	0.9300
S—C1	1.7721 (17)	C11—C12	1.378 (3)
S—C15	1.7996 (18)	C11—H11	0.9300
F—C4	1.365 (2)	C12—C13	1.379 (3)
O1—C7	1.375 (2)	C13—C14	1.380 (3)
O1—C8	1.3776 (19)	C13—H13	0.9300
C1—C8	1.374 (2)	C14—H14	0.9300
C1—C2	1.444 (2)	C15—C20	1.381 (3)
C2—C7	1.391 (2)	C15—C16	1.387 (2)
C2—C3	1.397 (2)	C16—C17	1.387 (3)
C3—C4	1.376 (3)	C16—H16	0.9300
C3—H3	0.9300	C17—C18	1.375 (4)
C4—C5	1.389 (3)	C17—H17	0.9300
C5—C6	1.384 (3)	C18—C19	1.388 (4)
C5—H5	0.9300	C18—H18	0.9300
C6—C7	1.382 (2)	C19—C20	1.390 (3)
C6—H6	0.9300	C19—H19	0.9300
C8—C9	1.455 (2)	C20—H20	0.9300
C9—C10	1.401 (2)		
			
O2—S—C1	107.56 (8)	C11—C10—H10	119.3
O2—S—C15	106.43 (8)	C9—C10—H10	119.3
C1—S—C15	96.72 (8)	C12—C11—C10	118.90 (18)
C7—O1—C8	106.98 (13)	C12—C11—H11	120.5
C8—C1—C2	107.73 (15)	C10—C11—H11	120.5
C8—C1—S	126.85 (13)	C11—C12—C13	121.57 (17)
C2—C1—S	125.42 (13)	C11—C12—Br	119.10 (14)
C7—C2—C3	119.78 (16)	C13—C12—Br	119.30 (14)
C7—C2—C1	104.55 (14)	C12—C13—C14	119.45 (18)
C3—C2—C1	135.66 (16)	C12—C13—H13	120.3
C4—C3—C2	115.72 (17)	C14—C13—H13	120.3
C4—C3—H3	122.1	C13—C14—C9	120.77 (18)
C2—C3—H3	122.1	C13—C14—H14	119.6
F—C4—C3	117.95 (18)	C9—C14—H14	119.6
F—C4—C5	117.67 (17)	C20—C15—C16	121.65 (18)
C3—C4—C5	124.38 (17)	C20—C15—S	118.49 (14)
C6—C5—C4	120.07 (17)	C16—C15—S	119.87 (15)
C6—C5—H5	120.0	C17—C16—C15	118.6 (2)
C4—C5—H5	120.0	C17—C16—H16	120.7
C7—C6—C5	116.00 (17)	C15—C16—H16	120.7
C7—C6—H6	122.0	C18—C17—C16	120.5 (2)
C5—C6—H6	122.0	C18—C17—H17	119.8
O1—C7—C6	124.94 (16)	C16—C17—H17	119.8
O1—C7—C2	111.03 (14)	C17—C18—C19	120.6 (2)
C6—C7—C2	124.03 (16)	C17—C18—H18	119.7
C1—C8—O1	109.69 (14)	C19—C18—H18	119.7
C1—C8—C9	135.70 (15)	C18—C19—C20	119.7 (2)
O1—C8—C9	114.61 (14)	C18—C19—H19	120.2
C10—C9—C14	117.93 (16)	C20—C19—H19	120.2
C10—C9—C8	119.97 (16)	C15—C20—C19	119.0 (2)
C14—C9—C8	122.10 (16)	C15—C20—H20	120.5
C11—C10—C9	121.35 (17)	C19—C20—H20	120.5
			
O2—S—C1—C8	−152.47 (14)	C7—O1—C8—C9	179.29 (13)
C15—S—C1—C8	97.90 (16)	C1—C8—C9—C10	−178.05 (18)
O2—S—C1—C2	28.34 (17)	O1—C8—C9—C10	1.2 (2)
C15—S—C1—C2	−81.29 (15)	C1—C8—C9—C14	2.0 (3)
C8—C1—C2—C7	−0.77 (18)	O1—C8—C9—C14	−178.71 (15)
S—C1—C2—C7	178.55 (12)	C14—C9—C10—C11	−0.5 (3)
C8—C1—C2—C3	178.37 (18)	C8—C9—C10—C11	179.57 (17)
S—C1—C2—C3	−2.3 (3)	C9—C10—C11—C12	−0.6 (3)
C7—C2—C3—C4	−0.9 (2)	C10—C11—C12—C13	1.5 (3)
C1—C2—C3—C4	−179.96 (18)	C10—C11—C12—Br	−176.61 (14)
C2—C3—C4—F	−179.75 (14)	C11—C12—C13—C14	−1.3 (3)
C2—C3—C4—C5	0.6 (3)	Br—C12—C13—C14	176.79 (15)
F—C4—C5—C6	−179.58 (15)	C12—C13—C14—C9	0.2 (3)
C3—C4—C5—C6	0.0 (3)	C10—C9—C14—C13	0.7 (3)
C4—C5—C6—C7	−0.4 (2)	C8—C9—C14—C13	−179.38 (17)
C8—O1—C7—C6	−179.14 (16)	O2—S—C15—C20	−13.32 (18)
C8—O1—C7—C2	0.74 (17)	C1—S—C15—C20	97.26 (17)
C5—C6—C7—O1	179.97 (15)	O2—S—C15—C16	166.15 (15)
C5—C6—C7—C2	0.1 (2)	C1—S—C15—C16	−83.27 (16)
C3—C2—C7—O1	−179.29 (14)	C20—C15—C16—C17	−2.5 (3)
C1—C2—C7—O1	0.02 (17)	S—C15—C16—C17	178.09 (15)
C3—C2—C7—C6	0.6 (2)	C15—C16—C17—C18	1.1 (3)
C1—C2—C7—C6	179.90 (16)	C16—C17—C18—C19	0.2 (3)
C2—C1—C8—O1	1.26 (18)	C17—C18—C19—C20	−0.3 (4)
S—C1—C8—O1	−178.05 (11)	C16—C15—C20—C19	2.4 (3)
C2—C1—C8—C9	−179.43 (17)	S—C15—C20—C19	−178.13 (17)
S—C1—C8—C9	1.3 (3)	C18—C19—C20—C15	−1.0 (4)
C7—O1—C8—C1	−1.24 (17)		

Symmetry codes: (i) −*x*+1, −*y*, −*z*+1.

### Hydrogen-bond geometry (Å, °)

**Table:**

Cg3 is the centroid of the C15–C20 phenyl ring.

**Table:**

*D*—H···*A*	*D*—H	H···*A*	*D*···*A*	*D*—H···*A*
C5—H5···Cg3^ii^	0.93	2.85	3.644 (3)	145

Symmetry codes: (ii) *x*, *y*+1, *z*.
